# Enhancing central blood pressure accuracy through statistical modeling: A proof-of-concept study

**DOI:** 10.3389/fcvm.2022.1048507

**Published:** 2022-11-23

**Authors:** Louis-Charles Desbiens, Nadir Goulamhoussen, Catherine Fortier, Amélie Bernier-Jean, Mohsen Agharazii, Rémi Goupil

**Affiliations:** ^1^Department of Medicine, Université de Montréal, Montréal, QC, Canada; ^2^Centre Hospitalier Universitaire (CHU) de Québec, Université Laval, Québec City, QC, Canada; ^3^Hôpital du Sacré-Cœur de Montréal, Montréal, QC, Canada

**Keywords:** central blood pressure, aortic blood pressure, invasive blood pressure, accuracy, brachial cuff-based, oscillometric devices for measurement of central BP, statistical modeling

## Abstract

**Background:**

Non-invasive estimation of central blood pressure (BP) may have better prognostic value than brachial BP. The accuracy of central BP is limited in certain populations, such as in females and the elderly. This study aims to examine whether statistical modeling of central BP for clinical and hemodynamic parameters results in enhanced accuracy.

**Methods:**

This study is a cross-sectional analysis of 500 patients who underwent cardiac catheterization. Non-invasive brachial cuff and central BP were measured simultaneously to invasive aortic systolic BP (AoSBP). Central BP was calibrated for brachial systolic (SBP) and diastolic BP (Type I calibration; C1SBP) or brachial mean and diastolic BP (Type II calibration; C2SBP). Differences between central SBP and the corresponding AoSBP were assessed with linear regression models using clinical and hemodynamic parameters. These parameters were then added to C1SBP and C2SBP in adjusted models to predict AoSBP. Accuracy and precision were computed in the overall population and per age or sex strata.

**Results:**

C1SBP underestimated AoSBP by 11.2 mmHg (±13.5) and C2SBP overestimated it by 6.2 mmHg (±14.8). Estimated SBP amplification and heart rate were the greatest predictors of C1- and C2-AoSBP accuracies, respectively. Statistical modeling improved both accuracy (0.0 mmHg) and precision (±11.4) but more importantly, eliminated the differences of accuracy seen in different sex and age groups.

**Conclusion:**

Statistical modeling greatly enhances the accuracy of central BP measurements and abolishes sex- and age-based differences. Such factors could easily be implemented in central BP devices to improve their accuracy.

## Introduction

Hypertension is the leading risk factor for global disease burden worldwide (1). It has been associated with numerous adverse health outcomes such as cardiovascular disease, dementia, and chronic kidney disease (2, 3), and is responsible for annual health costs of more than 130 billion dollars in the United States alone (4). Brachial cuff measurement of blood pressure (BP) has been used for more than a century to diagnose and guide the management of hypertension. While brachial BP is clinically convenient and was shown in a meta-analysis to closely approximate the true (invasively measured) aortic BP at the populational level (mean difference of 0.3 mmHg), it remains imprecise at the individual level (mean absolute differences of 8 mmHg) (5).

Since direct measurement of the true aortic BP is not feasible on a routine basis, non-invasive methods to estimate central BP have been developed. Non-invasive aortic BP (herein referred to as “central BP”) estimating devices use tonometry or cuff-based methods to capture a peripheral waveform that can be transformed into an estimated central waveform after calibration with either systolic BP (SBP) and diastolic BP (DBP; Type I calibration) or mean BP (MAP) and DBP (Type II calibration) (6, 7). Cuff-based methods can also be extended to 24 h monitoring to provide nocturnal and diurnal patterns of central BP (8, 9). While these non-invasive measurements of central BP have been found to be superior predictors of cardiovascular risk and end-organ damage compared to brachial BP (10–16), they are limited by a dependency from accurate brachial cuff BP measures which limits their routine clinical use. Both radial tonometry and brachial-cuff devices have indeed been shown to underestimate the true aortic SBP (AoSBP) by 5–8 mmHg when using Type I calibration (17, 18). Since this underestimation is not seen when invasive measurements of brachial BP are used for calibration (17), it was hypothesized that the use of brachial cuff SBP (which is known to underestimate the true brachial SBP) was partly responsible for the inaccuracy between central SBP and AoSBP (5). Type II calibration of central SBP, which does not rely on SBP but on MAP, was instead shown to be more accurate while slightly overestimating AoSBP (19).

Although the inaccuracy between central BP and the true aortic SBP was traditionally attributed to errors in the measurement of brachial cuff BP, recent studies conducted by our group have shown that several clinical characteristics (notably female sex and height) influenced the accuracy between non-invasive and invasive brachial BP (20, 21). Since central BP measurements are highly dependent on an accurate brachial cuff BP for calibration, we hypothesized that adjusting for such clinical parameters could enhance the performance of central BP devices. Therefore, using a cohort of patients with true aortic BP measurements made during cardiac catheterization, we aimed to (1) identify which clinical parameters are associated with the inaccuracy between central BP and true aortic BP measurements; (2) evaluate the performance of models based on these clinical parameters in the prediction of true aortic BP measurements; and (3) assess the impact of these clinical parameters on age and sex-associated differences in accuracy.

## Materials and methods

### Design, data sources, and population

Data from this study was derived from a prospective cohort of patients undergoing clinically indicated cardiac catheterization (for either suspected coronary artery disease or acute coronary syndromes) at the *Hôpital du Sacré-Coeur de Montréal* (Canada) between January and December 2019 (20). Patients were included unless: (1) they had previously documented severe aortic stenosis or atrial fibrillation (since their pulse wave characteristics may differ from the general population); (2) pre-catheterization inter-arm SBP/DBP difference was elevated (>10 mmHg); or (3) invasive and non-invasive BP measures could not be taken simultaneously for safety, time or technical reasons (notably if a patient urgently required coronary angiography due to its clinical condition). Clinical data was obtained by medical chart review. Written consent was obtained before inclusion in the study. Our study was conducted following the ARTERY Society task force consensus statement on protocol standardization (22) and was approved by the institutional ethics review board of the *CIUSSS du Nord-de-l’île-de-Montréal*. All investigators adhered to the Declaration of Helsinki.

### Invasive blood pressure measurement

True aortic BPs were measured in hemodynamically stable conditions at the end of the cardiac catheterization procedure by the cardiologist responsible for the intervention. Intra-arterial vasodilators were given after radial artery cannulation, but no vasoactive or contrast agent was given at least 5 min prior to measurements of all invasive BP. A fluid-filled catheter (5F or 6F) was connected to the *Xper Flex Cardio Physiomonitoring System* (Phillips, Amsterdam, Netherlands) with the manifold position maintained at heart level on the angiography table. Following the cardiac procedure, the catheter tip was positioned using fluoroscopy within 3 cm of the aortic valve in the aorta. Blood was then aspirated to remove bubbles and the catheter was flushed with 10 ml of 0.9% sodium chloride solution. The monitoring system was zeroed and calibrated before each measure. After the calibration of the system and visual confirmation of the correct waveform, BP measures were recorded for every heartbeat during 20 s and then averaged.

### Non-invasive blood pressure measurement

Non-invasive BP was measured in the arm contralateral to the arterial access using the Mobil-O-Graph NG device (I.E.M., Stolberg, Germany) with an appropriately sized cuff (7, 23). Trained research personnel were responsible for non-invasive BP measurements. The Mobil-O-Graph device first measures brachial cuff BP using a validated oscillometric procedure with stepwise deflation of the cuff. Then, it reinflates at a pressure below the brachial cuff DBP for 10 s to capture and generate a brachial pulse waveform. This waveform is then converted into an aortic waveform using a generalized transfer function. This conversion can either be calibrated for brachial SBP and DBP (Type I calibration; C1SBP) or mean BP and DBP (Type II calibration; C2SBP). Other aortic parameters {augmentation index corrected for 75 bpm [100 × (pulse pressure − incident pressure wave height) ÷ pulse pressure], reflection magnitude (backward ÷ forward component of the waveform), estimated aortic pulse wave velocity, SBP amplification (brachial cuff SBP − C1SBP)} can be derived using this waveform. The inflation sequence was timed such that invasive aortic and non-invasive BP measurements coincided.

### Statistical analysis

Analyses were conducted with R 4.1.0 (R Project for Statistical Computing). *p*-Values under 0.05 were considered significant. Categorical descriptive characteristics were presented as counts (percentages) and continuous characteristics as either means (±SD) or medians (interquartile range) according to their distribution. Accuracy was calculated as the mean difference between a *non-invasive/estimated* BP and the corresponding *invasive/true* BP and was assessed in both the overall population and in age and sex-strata (22). Differences between AoSBP and central SBP were modelized using linear regression models with pre-specified clinical characteristics (demographics, comorbidities, and hemodynamic parameters) as predictors and either C1SBP or C2SBP as outcome. The role of each predictor was assessed by computing its effect on the mean difference (the *beta* coefficient in the linear regression model), its *p*-value and its coefficient of determination (partial *R*^2^; computed by the *rsq* package). Global *R*^2^ and mean differences were also computed. Absence of multicollinearity was verified using variance inflation factors, which were all below 5.

The performance of central SBP either alone or with the addition of clinical and non-invasive hemodynamic parameters was assessed using linear regression models with AoSBP as an outcome. Successively tested models included C1SBP alone, C2SBP alone, C1SBP-C2SBP combined, and then four adjusted models including C1SBP and C2SBP plus clinical parameters: (1) a demographics model, using age, and sex; (2) a pulse wave analysis (PWA) model using the demographics model plus heart rate and PWA parameters (SBP amplification, augmentation index, reflection magnitude, and estimated aortic pulse wave velocity); (3) a full model using the parameters of the PWA model plus height, weight, eGFR, active smoking, diabetes, anti-hypertensive medications, statin use, and aspirin use; and (4) a model with parameters contained in the full but selected using a least absolute shrinkage and selection operator (LASSO) procedure. Performance was assessed using accuracy, precision (SD of the accuracy), goodness-of-fit (*R*^2^) and the percentage of patients with non-invasive SBP within ±10 mmHg of the corresponding AoSBP measurement. Models were also assessed after a bootstrap resampling procedure of 1,000 iterations. As sensitivity analysis, height and weight were replaced by body mass index in the fully adjusted model. The impact of adjustment for clinical parameters was also assessed by building modified Bland–Altman plots for both C1SBP and C2SBP either before or after adjustment (22). Finally, the impact of adjustment on age- (40–60, 60–80, and ≥80 years old) and sex-specific differences was calculated.

## Results

### Population characteristics

**FIGURE 1:**
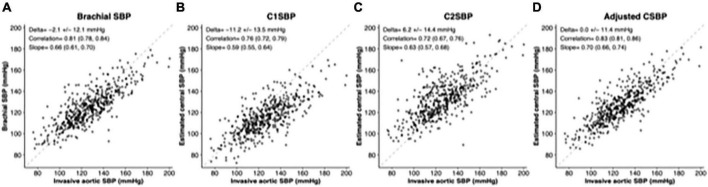
Scatter plots of brachial SBP/estimated central SBP with invasive aortic SBP. Each gray dot represents the relationship between invasive aortic SBP and brachial SBP/estimated central SBP for a given individual. Correlation coefficients, regression slopes and mean accuracy for aortic SBP are displayed with their 95th confidence interval. **(A)** Brachial SBP; **(B)** C1SBP; **(C)** C2SBP; and **(D)** adjusted central SBP (full model). SBP, systolic blood pressure.

Characteristics of the 500 participants included in this study are displayed in Table 1. The mean age was 65.7 ± 10.0 years and 71% were men. Most patients used at least one anti-hypertensive medication (79.8%), aspirin (70.8%), or a statin (69.6%). Mean brachial cuff SBP was 124.4 mmHg (±16.8) and mean brachial cuff DBP was 76.6 mmHg (±10.8). The mean true aortic SBP was 126.5 mmHg (±21.7) while mean C1SBP was 115.4 mmHg (±16.2) and C2SBP was 132.7 mmHg (±18.1). The distribution of C1SBP and C2SBP according to AoSBP are displayed in Figure 1.

**TABLE 1:** Population characteristics.

Clinical characteristics	*N* = 500
Sex, male	355 (71.0%)
Age	65.7 ± 10.0
Height (cm)	170.0 ± 9.8
Weight (kg)	82.7 ± 18.7
Body mass index (kg/m^2^)	28.5 ± 5.6
eGFR (ml/min/1.73 m^2^)	80.2 ± 17.4
Active smoking	124 (24.8%)
Dyslipidemia	321 (64.2%)
Hypertension	329 (65.8%)
Diabetes	141 (28.2%)
Prior stroke	11 (2.2%)
Peripheral artery disease	23 (4.6%)
Heart failure	49 (9.8%)
Positive coronary angiography procedure	332 (66.4%)
1 vessel disease	128 (25.6%)
2 vessels disease	122 (24.4%)
3 vessels disease	82 (16.4%)
**Medication**	
**Anti-hypertensive medication**	
Any	399 (79.8%)
RAS blockers	232 (46.4%)
Beta-blockers	278 (55.6%)
Diuretics	80 (16.0%)
Calcium channel blockers	131 (26.2%)
Aspirin	354 (70.8%)
Statin	348 (69.6%)
**Arterial parameters**	
Invasive aortic SBP (mmHg)	126.5 ± 21.7
Brachial cuff SBP (mmHg)	124.4 ± 16.8
Brachial cuff DBP	76.6 ± 10.8
C1SBP (mmHg)	115.4 ± 16.2
C2SBP (mmHg)	132.7 ± 18.1
Estimated SBP amplification (mmHg)	8.0 (5.0, 12.0)
Heart rate (bpm)	67.6 ± 12.1
Aortic pulse wave velocity (m/s)	9.39 ± 1.80
Reflection magnitude	66.9 ± 9.1
Augmentation index at 75 bmp	20.3 (10.6, 28.1)

Values are expressed as *n* (%), mean ± SD or median (interquartile range). Estimated SBP amplification is calculated by subtracting brachial cuff SBP and C1SBP. SBP, systolic blood pressure. C1SBP, Type I central BP obtained through calibration with brachial cuff SBP and diastolic blood pressure; C2SBP, Type II central BP obtained through calibration with brachial cuff mean and diastolic blood pressures; eGFR, glomerular filtration rate estimated using the CKD-EPI formula; SBP, systolic blood pressure; DBP, diastolic blood pressure.

### Accuracy of brachial and central systolic blood pressure

**FIGURE 2:**
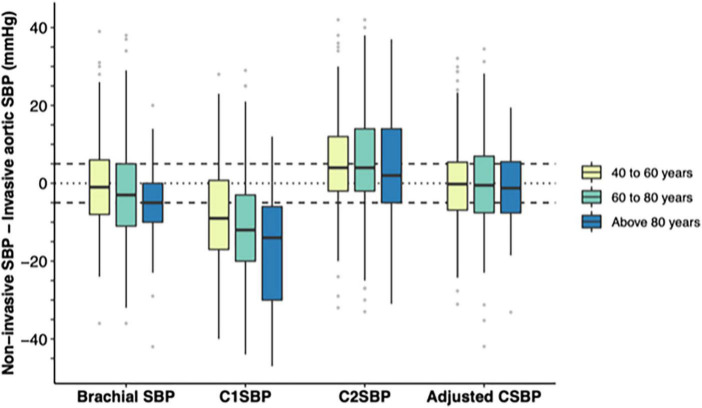
Differences between brachial SBP/estimated central SBP and invasive aortic SBP by age before and after adjustment. The distribution of differences by age strata are displayed by boxplots before and adjustment (full model). The horizontal black line in the middle of each box indicates the median, the square extremities indicate the interquartile range, and the vertical black lines end at either the minimum (first quartile minus 1.5 times the interquartile range) or the maximum (third quartile plus 1.5 times the interquartile range). Gray dots indicate outliers. The full models include the age, sex, heart rate, estimated SBP amplification, pulse wave velocity, augmentation index at 75 bpm, reflection magnitude, height, weight, eGFR, active smoking, diabetes, and antihypertensive treatment. C1SBP, Type I calibrated central SBP; C2SBP, Type II calibrated central SBP; eGFR, estimated glomerular filtration rate; SBP, systolic blood pressure.

**FIGURE 3:**
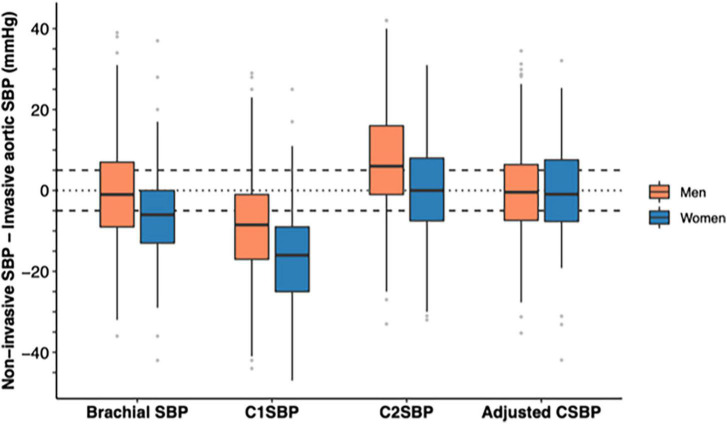
Differences between brachial SBP/estimated central SBP and invasive aortic SBP by sex before and after adjustment. The distribution of differences by sex strata are displayed by boxplots before and adjustment (full model). The horizontal black line in the middle of each box indicates the median, the square extremities indicate the interquartile range, and the vertical black lines end at either the minimum (first quartile minus 1.5 times the interquartile range) or the maximum (third quartile plus 1.5 times the interquartile range). Gray dots indicate outliers. The full models include the age, sex, heart rate, estimated SBP amplification, pulse wave velocity, augmentation index at 75 bpm, reflection magnitude, height, weight, eGFR, active smoking, diabetes, antihypertensive treatment. C1SBP, Type I calibrated central SBP; C2SBP, Type II calibrated central SBP; eGFR, estimated glomerular filtration rate; SBP, Systolic blood pressure.

As displayed in Table 2, brachial cuff SBP was 2.1 mmHg lower than AoSBP (with precision, or SD, of ±12.1) while C1SBP underestimated AoSBP by 11.2 mmHg (±13.5) and C2SBP overestimated AoSBP by 6.2 mmHg (±14.8). When these differences were stratified by age and sex (Table 2 and Figures 2, 3), the underestimation of AoSBP by brachial SBP and C1SBP was more pronounced in older patients and in women. Similarly, the overestimation of AoSBP by C2SBP was more pronounced in younger patients and in men. These differences were statistically significant for age (brachial SBP and C1SBP) and for sex (brachial SBP, C1SBP, and C2SBP).

**TABLE 2:** Differences between estimated and invasive aortic SBP by age and sex.

	Brachial SBP	C1SBP	C2SBP	Adjusted CSBP
Overall	−2.1 ± 12.1	−11.2 ± 13.5	6.2 ± 14.4	0.0 ± 11.4
**Age**				
40–60 years	0.1 ± 12.6	−8.3 ± 12.9	7.2 ± 15.3	−0.1 ± 11.5
60–80 years	−2.7 ± 11.7	−11.9 ± 13.2	6.0 ± 14.6	0.1 ± 11.4
Above 80 years	−5.7 ± 12.8	−16.0 ± 16.6	3.8 ± 14.1	−1.0 ± 11.7
*p*-Value	0.015	0.003	0.453	0.859
**Sex**				
Men	−0.3 ± 11.7	−8.7 ± 12.9	8.5 ± 13.9	0.0 ± 11.3
Women	−6.5 ± 12.1	−17.3 ± 13.1	0.6 ± 15.3	0.0 ± 11.8
*p*-Value	<0.001	<0.001	<0.001	1.000

Differences between estimated and invasive aortic SBP in each age and sex strata are presented as mean difference ± SD. The adjusted model presented is the *fully adjusted model*, which includes age, sex, heart rate, estimated SBP amplification, pulse wave velocity, augmentation index at 75 bpm, reflection magnitude, height, weight, eGFR, active smoking, diabetes, antihypertensive treatment, statin use, and aspirin use. p-Values were computed using one-way ANOVA and Student’s t-test. C1SBP, Type I central systolic blood pressure; C2SBP, Type II systolic blood pressure; CSBP, central systolic blood pressure.

### Predictors of central systolic blood pressure inaccuracy

As shown in Supplementary Table 1, 27.9% of the mean difference between C1SBP and AoSBP could be explained by clinical parameters contained in a linear regression model. Higher SBP amplification significantly increased the underestimation of AoSBP by C1SBP and was the factor with the most elevated partial *R*^2^ in the model (10.1%). Other parameters significantly associated with mean differences between C1SBP and AoSBP were height (partial *R*^2^ = 1.8%), age (1.1%) and augmentation index at 75 bpm (0.9%).

A slightly smaller share of the mean differences between C2SBP and AoSBP (25.2%) was explained by clinical parameters contained in a linear regression model (Supplementary Table 2). Patients with lower heart rate had higher levels of overestimation (+4.7 mmHg per 10 bpm decrease in heart rate). Besides heart rate (partial *R*^2^ = 11.8%), augmentation index at 75 bpm (1.4%), age (1.1%) and estimated SBP amplification (0.8%) were significantly associated with mean differences between C2SBP and AoSBP.

### Impact of adjustment on the accuracy and precision of non-invasive systolic blood pressure

Table 3 displays the impact of various levels of adjustment on the prediction of AoSBP by estimated central SBP. By design, all models yielded accuracies of 0.0 mmHg. Compared to either C1SBP or C2SBP alone, combining both increased precision, model *R*^2^ and the percentage of patients with predicted SBP between ±10 mmHg of the AoSBP. Similarly, successively adding clinical characteristics also increased predictive performance. The full model, adjusting for all clinical and hemodynamic characteristics with both C1SBP and C2SBP, resulted in the best enhancement of central BP with a precision of 11.4 mmHg, a model *R*^2^ of 69.6 and 66.6% of patients within 10 mmHg of the reference invasive value. This performance was higher than the one observed for brachial SBP. A simpler model with characteristics chosen by a LASSO procedure (C1SBP, C2SBP, age, sex, estimated SBP amplification, augmentation index at 75 bpm, and height) resulted in similar predictive characteristics while containing a much smaller number of predictors. Interestingly, most characteristics identified by the LASSO procedure were the ones significantly associated with mean differences between crude central SBP estimates and AoSBP. Similar findings concerning accuracy, precision, *R*^2^ and patients within 10 mmHg were obtained after a bootstrap procedure using 1,000 resamples. Sensitivity analyses replacing height and weight by body mass index in the fully adjusted model yielded similar accuracy (difference of 0 mmHg), precision (11.5 mmHg), *R*^2^ (69.0) and percentage of patients within 10 mmHg of reference value (67.2%).

**TABLE 3:** Prediction of invasive aortic SBP by estimated central SBP and clinical parameters.

	Overall cohort (*n* = 500)				Bootstrap validation (1,000 replications)			
	Accuracy (mmHg)	Precision (mmHg)	*R* ^2^	±10 mmHg (%)	Accuracy (mmHg)	Precision (mmHg)	*R* ^2^	±10 mmHg (%)
Brachial SBP	–2.1	12.1	65.7	62.6	–2.1	12.1	65.7	62.6
C1SBP	–11.2	13.5	57.7	56.4	–11.2	13.5	57.7	56.0
C2SBP	6.2	14.4	51.6	55.4	6.2	14.4	51.6	54.6
**Adjusted SBP**								
C1 + C2-SBP	0.0	13.0	60.8	59.0	0.0	13.0	60.7	58.5
Demographics model	0.0	12.3	64.9	62.0	0.0	12.4	64.6	60.9
PWA model	0.0	11.6	68.7	65.0	0.0	11.7	68.1	64.0
Full model	0.0	11.4	69.6	66.6	0.0	11.7	68.5	64.7
LASSO model	0.0	11.6	68.9	65.8	0.0	11.7	68.5	64.8

Accuracy is expressed as the mean difference between estimated and invasive aortic SBP. Precision is expressed as the standard deviation of the difference between estimated and invasive aortic SBP. *R*^2^ represent the proportion of the variance of the invasive aortic SBP explained by the independent variables. *Demographics model*: age, sex, C1SBP, and C2SBP. PWA model: demographics model + heart rate, estimated SBP amplification, pulse wave velocity, augmentation index at 75 bpm, and reflection magnitude. Full model: PWA model + height, weight, eGFR, active smoking, diabetes, antihypertensive treatment, statin use, and aspirin use. *LASSO model*: age, sex, C1SBP, C2SBP, estimated SBP amplification, augmentation index at 75 bpm, and height. C1SBP, Type I calibrated central SBP; C2SBP, Type II calibrated central SBP; eGFR, estimated glomerular filtration rate; LASSO, least absolute shrinkage and selection operator; PWA, pulse wave analysis; SBP, systolic blood pressure.

**FIGURE 4:**
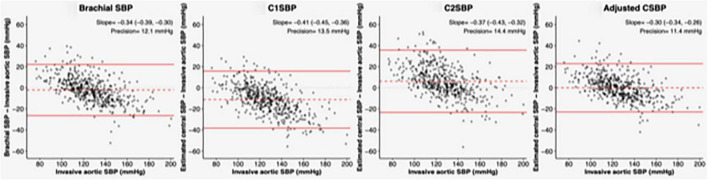
Bland–Altman plots for aortic SBP before and after adjustment. Modified Bland–Altman plots show the reference intra-arterial measurements (aortic SBP) on the *X*-axis instead of the mean of both measurements. Left to right panels show in order the agreement of brachial cuff SBP, C1SBP, and C2SBP with aortic SBP. All values are in mmHg. Red dashed lines and red solid lines represent mean differences (bias) and limits of agreement (bias ± 2 SDs) respectively. C1SBP, Type I central systolic blood pressure; C2SBP, Type II systolic blood pressure; SBP, systolic blood pressure.

The impact of adjustment for clinical characteristics was also assessed by building modified Bland–Altman plots for brachial SBP, C1SBP, C2SBP, and full model-adjusted central SBP. As shown in Figure 4, the underestimation of AoSBP by crude non-invasive SBP (brachial SBP, C1SBP, and C2SBP) was more pronounced at higher values, yielding negative Bland–Altman slopes. Adjustment for previously stated characteristics led to the smallest slope of Bland–Altman plots (slope = −0.30 [−0.34, −0.26]) among the four non-invasive parameters evaluated.

Finally, adjusting for clinical characteristics eliminated the age- and sex-associated differences in accuracy between CSBP and AoSBP, as shown in Table 2 and Figures 2, 3. Resulting *p*-values for age and sex differences were respectively of 0.859 (age) and 1.000 (sex).

## Discussion

In our cohort of 500 patients with simultaneous invasive and non-invasive BP measurements, we identified several parameters that contributed to the accuracy and precision of central BP measurements toward the true (invasive) aortic SBP. We then showed that by adjusting central SBP measurements with clinical and non-invasive hemodynamical characteristics, the predictive performance of central BP can be significantly enhanced and that inaccuracies inherent to age and sex can be eliminated.

In this study, we first observed that Type I calibration central BP underestimated AoSBP while Type II calibration central BP overestimated it. These findings were comparable to previous studies (17–19), but the underestimation of AoSBP by C1SBP in our study was larger in magnitude than the one observed in a previous study validating the Mobil-O-Graph device. Nevertheless, this degree of underestimation is in line with the range of differences observed in a previous meta-analysis of studies using tonometer-based devices, in which underestimations as high as 15 mmHg have been reported. Although the gap between true aortic and central SBP has been traditionally attributed to imprecisions in the brachial cuff BPs used for calibration, we aimed to evaluate whether clinical characteristics might be linked to accuracy. We observed that elevated estimated SBP amplification was the strongest determinant of the underestimation of AoSBP by C1SBP. Since this parameter is computed by subtracting C1SBP from brachial cuff SBP, it was expected that patients with lower C1SBP (and therefore higher estimated SBP amplification) would have greater levels of underestimation (24). Nevertheless, we observe that a parameter solely derived from estimated values could identify patients with a greater inaccuracy between estimated and invasive AoSBP. In contrast, we observed that low heart rate was the strongest determinant of the overestimation of AoSBP by C2SBP. Again, this finding can be explained by the changes in arterial waveform shape at lower heart rates (notably an earlier reflected wave) which decreases the peripheric to central BP ratio and could lead to overestimation of aortic SBP (25). In addition to these two parameters, we showed that higher age and augmentation index were associated with increased underestimation of AoSBP by both C1SBP and C2SBP. This relationship may reflect the greater loss of peripheral pulse pressure amplification seen in patients with high aortic stiffness (associated with age and the augmentation index) that is not fully accounted for by the generalized transfer function (26–29).

According to the ARTERY Society guidelines (22), an accurate central BP device should have an overall accuracy within 5 mmHg and a precision within 8 mmHg compared to reference invasive measurements. These thresholds of accuracy and precision have nevertheless not been met in most previous validation studies using tonometry or cuff-based devices, which still limits the clinical application of central BP (17–19). In our study, neither the two calibrations of central SBP nor usual brachial SBP did fulfill the ARTERY standards relative to invasive aortic SBP. Hence, adjusted models using clinical and hemodynamic parameters were tested. Compared to unadjusted models, models adding PWA and clinical parameters to central SBP led to a mean accuracy of 0 mmHg in the global and in the age and sex-stratified populations. While this finding was expected with statistical modeling, a similar accuracy was observed even after bootstrap validation, thus reinforcing our findings concerning the role of adjustment on central SBP accuracy. Furthermore, adjustment for these parameters also increased the goodness-of-fit (as demonstrated by the increase of the calculated *R*^2^), the precision (or SD) and the percentage of patients within 10 mmHg of the invasive aortic SBP in the overall population. These results are in line with the recent work of Esposito et al., who studied 93 patients undergoing cardiac catheterization and reported that the 6.4 mmHg inaccuracy (6.4 mmHg) and precision (17.4 mmHg) between CSBP and AoSBP were improved after the use of a linear-fit function (30). However, in contrast with our study, these authors did not include any clinical or hemodynamical parameters in their equation (only a slope and an intercept), did not study C1SBP and C2SBP separately, used SphygmoCor rather than the Mobil-O-Graph, and had a smaller sample size than ours.

It has previously been shown that at similar brachial SBPs, women appear to have higher CVD risk (31, 32). Hypotheses such as smaller vessel caliber or more pronounced aging-related loss of compliance have been proposed to explain this association (33, 34). As such, our team has recently shown that female sex is associated with a greater inaccuracy of brachial cuff, C1SBP for AoSBP while male sex is associated with greater inaccuracy of C2SBP for predicting AoSBP (20, 21). As expected, we obtained similar findings in this study using the same cohort. However, we expanded this previous work by showing that differences in accuracy were also observed for age in both brachial SBP, C1SBP, and C2SBP. These differences were easily eliminated by adding clinical and hemodynamical confounders to C1SBP and C2SBP. In short, statistical modeling erased the overall inaccuracy of both Type I and Type II central SBP, removed age- and sex-related differences in accuracy, and slightly improved other predictive parameters.

Interestingly, we also showed that a model containing only variables selected by LASSO led to an improvement similar to our fully adjusted model, but with much less complexity. This simplified model includes easily obtained characteristics (age, sex, and height) and variables automatically calculated by the Mobil-O-Graph (Type I and II central SBPs, estimated SBP amplification and augmentation index at 75 bpm) which could facilitate its implementation. Nevertheless, while all predictive parameters were improved by adjustment, none of the obtained models had a precision ≤ 8 mmHg when compared to invasive measurements, hence not adhering to the precision criteria as per ARTERY guidelines (22). This observation is coherent with the fact that only 25–28% of the invasive-estimated inaccuracy was explained by clinical parameters, thus leading to only a relatively small improvement in precision after adjustment. Our findings thus reinforce the major role of inter-individual differences in pulse waveforms across the arterial tree in central BP estimation.

Our study has several strengths. To our knowledge, it is the first study to evaluate the effect of statistical modeling on the predictive performance of non-invasive central BP estimates. Second, we used a robust methodology following the ARTERY guidelines to obtain invasive BP measurements (22). Third, non-invasive measurements were standardized by research personnel and taken simultaneously to the invasive aortic BP measurements. Furthermore, several clinical and hemodynamic parameters were used for model adjustment. Lastly, the effect of adjusted models was assessed using several statistical and graphical methods. However, some limitations are worth considering. Our results are based on the Mobil-O-Graph device and as such, may not be generalizable to other central BP devices. Also, as all patients required cardiac catheterization for a clinical indication, it remains to be determined if our findings are generalizable to patients without suspected cardiac disease, although a large-scale invasive BP study on other populations is not ethically feasible. The exclusion of participants with inter-arm SBP differences above 10 mmHg also limits the external validity of our results. Finally, external validation will be required before the models developed in this study could be adopted at a larger scale.

## Conclusion

Using a large cohort of patients with simultaneously measured invasive and non-invasive BP, we showed that both brachial SBP and non-invasive central SBP have sex- and age-associated differences in accuracy. We identified easily accessible clinical factors associated with non-invasive SBP accuracy and showed that adjusting central SBP measurements with these parameters improves the accuracy and precision toward the true aortic SBP. More so, we succeeded in removing the inaccuracy bias inherent to advancing age and female sex. These findings pave the way to an enhancement of central BP measurements that could potentially minimize under- and overtreatment of hypertension. Integration of such parameters into the estimating algorithms of central BP may not only improve accuracy, but also constitutes a first step toward a personalized approach for hypertension diagnosis and treatment targets.

## Data availability statement

The raw data supporting the conclusions of this article will be made available by the authors, without undue reservation.

## Ethics statement

The studies involving human participants were reviewed and approved by the Comité d’Éthique de la Recherche du CIUSSS du Nord-de-l’île-de-Montréal. The patients/participants provided their written informed consent to participate in this study.

## Author contributions

RG was responsible for the conception and design of the study, and oversaw all aspect of the study and manuscript. L-CD and NG performed the statistical analysis. NG wrote the first draft of the manuscript. L-CD wrote the second draft of the manuscript. CF, AB-J, and MA provided critical appraisal of the study conception, statistical analysis plan, and manuscript. All authors contributed to manuscript revision, read, and approved the submitted version.
