# Supplementary material for: Enhancing central blood pressure accuracy through statistical modeling: A proof-of-concept study
Source: Front Cardiovasc Med. 2022 Nov 23;9:1048507. doi: 10.3389/fcvm.2022.1048507 (PMC9728538; doi:10.3389/fcvm.2022.1048507)
Supplement: Supplementary file 1 [file Data_Sheet_1.PDF]

TABLE S1. Variation between Type I calibration non-invasive central SBP and true (invasive) aortic SBP according to clinical parameters

	**Mean difference (mmHg)**	***P-value***	**R^2 (%)**
**Overall**	**-11.2 ± 13.5**		
**Clinical parameters**			**27.9**
Estimated SBP amplification (per 1 mmHg)	-0.8 (-0.9, -0.5)	< 0.001	10.1
Height (per 10 cm)	2.5 (0.8, 4.1)	0.003	1.8
Age (per 10 years)	-1.6 (-3.0, -0.3)	0.019	1.1
Augmentation index @ 75 bpm (per 5)	-0.6 (-1.2, -0.1)	0.032	0.9
Sex (Men)	2.8 (-0.4, 6.1)	0.086	0.6
Heart rate (per 10 bpm)	0.9 (-0.2, 1.9)	0.099	0.6
Reflection magnitude (per 1)	0.1 (0.0, 0.2)	0.171	0.4
Weight (per 10 kg)	-0.4 (-1.1, 0.3)	0.239	0.3
eGFR (per 10 ml/min/1.73m^2)	-0.4 (-1.1, 0.3)	0.265	0.3
Diabetes	-0.7 (-3.1, 1.7)	0.557	0.1
Antihypertensive treatment	-0.8 (-3.5, 2.0)	0.582	0.1
Active smoking	-0.7 (-3.2, 1.9)	0.610	0.1
Statin use	0.4 (-2.0, 2.7)	0.766	0.0
Aspirin use	-0.2 (-2.7, 2.3)	0.852	0.0

Mean differences, *p-values* and R^2 were computed from a linear regression model using the difference between Type I calibration non-invasive central SBP and true (invasive) aortic SBP as outcome. Partial R^2 are displayed except for the overall value, which is the global R^2 of the model. SBP, Systolic blood pressure; eGFR, estimated glomerular filtration rate.

TABLE S2. Variation between Type II calibration non-invasive central SBP and true (invasive) aortic SBP according to clinical parameters

	**Mean difference (mmHg)**	***P-value***	**R^2 (%)**
**Overall**	**6.2 ± 14.8**		
**Clinical parameters**			**25.2**
Heart rate (per 10 bpm)	-4.7 (-5.8, -3.5)	< 0.001	11.8
Augmentation index @ 75 bpm (per 5)	-0.9 (-1.5, -0.2)	0.009	1.4
Age (per 10 years)	-1.8 (-3.3, 0.0)	0.019	1.1
Estimated SBP amplification (per 1 mmHg)	0.2 (0.0, 0.4)	0.046	0.8
Height (per 10 cm)	1.7 (-0.2, 0.4)	0.076	0.6
Antihypertensive treatment	-2.1 (-5.1, 1.0)	0.181	0.4
eGFR (per 10 ml/min/1.73m^2)	-0.5 (-1.3, 0.3)	0.201	0.3
Sex (Men)	1.9 (-1.8, 5.5)	0.312	0.2
Active smoking	-1.3 (-4.2, 1.6)	0.372	0.2
Diabetes	0.5 (-2.2, 3.2)	0.714	0.0
Weight (per 10 kg)	0.1 (-0.6, 0.9)	0.746	0.0
Aspirin use	0.3 (-2.5, 3.1)	0.823	0.0
Statin use	0.1 (-2.5, 2.8)	0.920	0.0
Reflection magnitude (per 1)	0.0 (-0.1, 0.1)	0.970	0.0

Mean differences, *p-values* and R^2 were computed from a linear regression model using the difference between Type II calibration non-invasive central SBP and true (invasive) aortic SBP as outcome. Partial R^2 are displayed except for the overall value, which is the global R^2 of the model. SBP, Systolic blood pressure; eGFR, estimated glomerular filtration rate.
